# Life-course Psychosocial Adversity and Biological Aging in the Hispanic Community Health Study / Study of Latinos: A Life-course Model Analysis

**DOI:** 10.21203/rs.3.rs-8725187/v1

**Published:** 2026-02-13

**Authors:** Yinxian Chen, Sarina Abrishamcar, Christian K. Dye, Maria M. Llabre, Linda C. Gallo, Krista M. Pereira, Martha Daviglus, Maria Argos, Jianwen Cai, Bharat Thyagarajan, Andrea Baccarelli, Carmen R. Isasi, Karen N. Conneely, Anke Huels, Timothy Lash, Shakira F. Suglia

**Affiliations:** 1 Department of Epidemiology, Rollins School of Public Health, Emory University, Atlanta, GA, USA; 2 Department of Anatomy, Biochemistry and Physiology, John A. Burns School of Medicine, University of Hawaii at Manoa, Honolulu, HI, USA; 3 Department of Psychology, University of Miami, Coral Gables, FL, USA; 4 Department of Psychology, San Diego State University, San Diego, CA, USA; 5 Department of Social Medicine, The University of North Carolina School of Medicine, Chapel Hill, NC, USA; 6 Institute for Minority Health Research, College of Medicine, University of Illinois at Chicago, Chicago, IL, USA; 7 Department of Environmental Health, Boston University School of Public Health, Boston, MA, USA; 8 Department of Biostatistics, The University of North Carolina at Chapel Hill, Chapel Hill, NC, USA; 9 Department of Laboratory Medicine and Pathology. University of Minnesota, Minneapolis, MN; 10 Department of Environmental Health, Harvard T.H. Chan School of Public Health, Boston, MA, USA; 11 Department of Epidemiology and Population Health, Albert Einstein College of Medicine, Bronx, NY, USA; 12 Department of Human Genetics, School of Medicine, Emory University, Atlanta, GA, USA; 13 Gangarosa Department of Environmental Health, Rollins School of Public Health, Emory University, Atlanta, GA, USA; 14 Department of Biostatistics and Bioinformatics, Rollins School of Public Health, Emory University, Atlanta, GA, USA

**Keywords:** psychosocial adversity, lifetime, biological aging, life-course model

## Abstract

Psychosocial adversity over the life course may impact the aging process. However, life-course models have yet to fully explain the biological embedding of psychosocial adversity in aging. A subsample of participants from the Hispanic Community Health Study/Study of Latinos with DNA methylation (DNAm) data (N=970) was used to evaluate the effect of adversity on biological aging and the most compatible life-course model. Epigenetic age was estimated from GrimAge and DunedinPACE. We modified a current Bayesian life-course model to estimate the effect of adversity from childhood to adulthood on epigenetic age acceleration and the weights contributed by childhood (*W*_*childhood*_) and adulthood (*W*_*adulthood*_), which sum to one. The model was also used to evaluate the compatibility with the sensitive period (*W*_*childhood*_ ≠ *W*_*adulthood*_) and accumulation models (*W*_*childhood*_ ≈ *W*_*adulthood*_). Causal mediation analysis assessed the pathway model by estimating the indirect effect of childhood adversity through adulthood adversity. Per-unit increase in adversity was associated with 0.91 years (95% credible interval [CrI]: 0.28, 1.53; *W*_*adulthood*_ = 82%, 95% CrI: 36%, 99%) increased GrimAge acceleration (AgeAccelGrim) and 0.013 years/calendar year (95% CrI: −0.005, 0.032; *W*_*childhood*_ = 49%, 95% CrI: 3%, 97%; *W*_*adulthood*_ = 51%, 95% CrI: 3%, 97%) increased DunedinPACE. A pronounced indirect effect of childhood adversity was found in AgeAccelGrim (0.23 years, 95% CI: 0.09, 0.37) but minimal in DunedinPACE (0.003 years/calendar year, 95% CI: −0.001, 0.006). Psychosocial adversity from childhood to adulthood may affect biological aging, with distinct life-course models explaining its effects on different aging markers.

## Introduction

Psychosocial adversity is prevalent among the US population across life stages. More than 60% of US adults report experiencing at least one adverse childhood experience (ACE), such as abuse, neglect, and household dysfunction, and about 20% report experiencing four or more adversities.^1^ In adulthood, many people in the US face different types of psychosocial adversity. More than 40% of adults report experiencing at least one type of intimate partner violence (e.g., sexual violence, stalking, and physical violence).^2^ Material deprivation has been reported as one of the most significant sources of stress in adults,^3^ and its latest estimated national prevalence is 37%.^4^ As a critical public health issue, substance abuse has affected 48.5 million people, which imposes substantial stress on their household members and friends.^5^

Psychosocial adversity can impose substantial stress on people and has been found to increase the risk of multiple chronic conditions, including cardiovascular disease (CVD),^6, 7^ diabetes,^8, 9^ and accelerated cognitive decline.^10, 11^ One potential mechanism could be the acceleration of biological aging (i.e., the gradual decline in function and tissue/organ integrity over time).^12, 13^ Epigenetic age has been increasingly used to reflect biological age more accurately than chronological age. Epigenetic age acceleration (EAA), the difference between epigenetic and chronological age, independently predicts mortality and morbidity,^14^ and is associated with childhood^15–17^ and adulthood adversity.^18–21^ Therefore, effective interventions to prevent the occurrence of adversities or their subsequent adverse impact could promote healthy aging and prevent or delay the onset of chronic diseases.

Most current studies focus on adversity in childhood or adulthood separately in relation to biological aging, and only a few consider the effect of adversity from a life-course perspective.^22–24^ Many psychosocial adverse conditions occur repeatedly across the lifespan,^25^ and adversities at different times could impact aging through differential mechanisms. For example, ACEs can alter a person’s development trajectory and persistently affect aging.^26, 27^ In contrast, exposure to adversities in adulthood could directly impair the ongoing aging organ systems or induce unhealthy lifestyles and, thus, accelerate biological aging.^28–30^ However, the underlying life-course model explaining the effect of adversity on aging is unclear. Childhood and adulthood could be independent exposure windows, and their impacts could be equal (i.e., accumulation model) or differ, with one being more vulnerable to adversity than the other (i.e., sensitive period model).^25, 31^ Additionally, adversity could be a chain exposure such that childhood adversity affects aging partially or fully by increasing the likelihood of adulthood adversity,^32^ which yields a disadvantageous aging trajectory compared to those without exposure in childhood (i.e., pathway model).^25, 31^

Without capturing the life-course pattern of adversity affecting biological aging, we are limited in identifying targets and timings for interventions. Studies focusing on adult adversities that do not consider the role of childhood adversity could also be vulnerable to left censoring, as the observed association between adulthood adversity could be confounded by childhood adversity, but this confounding is uncontrolled because of missing childhood data.^33^ Given the value of informing target interventions on aging and subsequent disease progression, it is warranted to estimate the effect of adversity on biological aging throughout the life-course and uncover the underlying pattern.

In this study, we estimated the effect of psychosocial adversity from childhood to adulthood on biological aging, estimated by epigenetic age, among a sample of adults from the Hispanic Community Health Study / Study of Latinos (HCHS/SOL). We evaluated four types of life-course models that could explain the observed data: sensitive period, critical period, accumulation, and pathway models. We proposed a step-by-step pipeline to identify the most compatible model explaining the effect of adversity on biological aging using an updated Bayesian life-course model and causal mediation analysis.

## Methods

### Study population

The data of the current study are from the HCHS/SOL, a community-based cohort of US Hispanic/Latino adults. Details of the study design and recruitment have been previously described.^34, 35^ Briefly, 16,415 Hispanic/Latino adults with diverse Hispanic/Latino backgrounds were recruited from four major US cities (Bronx, NY, Chicago, IL, Miami, FL, and San Diego, CA). Participants were eligible for inclusion if they were not currently pregnant, spoke English or Spanish, and self-identified as Hispanic/Latino. The baseline examination (Visit 1) was conducted during 2008–2011. Among HCHS/SOL Visit 1 participants, 5,313 were also enrolled in the Sociocultural Ancillary Study (SCAS) and asked to complete the comprehensive psychosocial assessment within nine months of their baseline exam. The second in-person HCHS/SOL examination (Visit 2) was conducted between 2014–2017, approximately six years after Visit 1, with repeated key measurements from Visit 1.

For this study, we randomly sampled 1000 participants who completed the SCAS and both HCHS/SOL Visits 1 and 2. DNA methylation (DNAm) data were measured at Visit 2. Generally, the characteristics of the sample were similar to the total HCHS/SOL population, although the participants in the subsample were slightly younger (Table S1). All participants in the final analysis consented to the use of their samples for DNA analyses. Finally, 970 participants with DNAm data at Visit 2 were included in the analysis. The study was approved by the Institutional Review Board of Emory University.

### Life-course Adversity

Four adverse events measured in both childhood and adulthood in HCHS/SOL were included: substance abuse by someone close, material deprivation, physical abuse, and sexual abuse (Table S2). Briefly, the included adversity types were self-reported via psychometrically validated tools, including the ACE study questionnaire,^36^ Traumatic Stress Inventory,^37^ and the Chronic Stress Scale,^38^ or single items. Each type of adversity was encoded as a binary variable (yes, no). Due to the low reported prevalence of physical and sexual abuse in adulthood (Table S3), we combined them into a single variable in each period: abuse (experiencing physical or sexual abuse, none). The primary exposures of interest were the cumulative score of childhood and adulthood adversity (i.e., cumulative adversity), with each summing all adverse events within the corresponding period (score ranged 0–3). We also considered each type of adverse event at different periods.

### DNA Methylation and Epigenetic Age

DNAm was measured from whole blood at Visit 2 using the Illumina Infinium HumanMethylationEPIC (850K) BeadChip (Illumina, Inc, San Diego, CA). We performed the preprocessing and normalization on raw IDAT files using the *minfi* package in R (version 4.3.2)^39^. Samples with a non-significant mean probe intensity detection p-value (p>0.01) were removed. This resulted in the removal of one failed sample; the matched timepoint for this sample was also removed after our final normalization step prior to downstream analyses. We then performed background correction and dye-bias normalization using the within-array normalization single sample “noob” (normal-exponential out-of-band).^40^ Normalized samples were then filtered at the probe level, removing unreliable CpGs based on insignificant probe intensities (detection p-value >0.01) in one or more samples, probes on sex chromosomes, probes covering known SNP sequences, and cross-reactive probes. Probe design type biases were corrected by using the Regression on Correlated Probes (RCP) method in the *ENmix* package in R.^41^ Using the Houseman’s cell-type deconvolution method based on DNAm data,^42^ we estimated blood cell-type proportions (B cell, NK cell, CD4+ T cell, CD8+ T cell, monocyte, and neutrophil) using the *ewastools* package (https://github.com/hhhh5/ewastools) with the reference dataset specific for peripheral blood cell types assayed on the 850K, provided by Salas et al.^43^ Finally, we obtained methylation β-values (i.e., the proportion of methylated signal in total signals), resulting in DNAm proportions ranging from 0 (unmethylated) to 1 (fully methylated). After sample and probe filtering (Supplemental Methods), 970 samples with DNAm data at Visit 2 were included in the following analysis.

We estimated biological aging using epigenetic age by two separate measures, the second-generation epigenetic clock, GrimAge^44^, and, DunedinPACE, a biomarker for the pace of biological aging.^45^ Details of these two measures are shown in the Supplemental Methods. GrimAge and DunedinPACE capture different aspects of aging. GrimAge was trained to predict chronological age and excess mortality risk beyond normal aging. It has been shown to better predict morbidity and mortality compared with first-generation clocks.^46^ GrimAge was calculated by uploading the DNAm data to the publicly available calculator (https://dnamage.genetics.ucla.edu) with the ‘normalization’ and ‘advanced analysis in blood’ options selected from a subset of probes with accompanying age, sex, and tissue information. EAA for GrimAge (AgeAccelGrim) was calculated by regressing GrimAge on chronological age and defined as the corresponding residual. A positive AgeAccelGrim indicates that the epigenetic age of an individual is older than expected given their chronological age. In contrast, DunedinPACE represents the deviation from the normal speed of physiological decline across body systems (i.e., biological aging) per calendar year. DunedinPACE was calculated in R following the procedures described in Belsky et al. using the DunedinPACE R package (https://github.com/danbelsky/DunedinPACE). The interpretation of the pace of aging differs from AgeAccelGrim. With reference to an average rate of 1 biological year per calendar year (year/calendar year), values >1 indicate a faster pace of biological aging per calendar year and values <1 indicate slower aging.

### Covariates and Confounding Assessment

Covariates were selected based on the hypothetical causal structure between exposure and outcome shown by a direct acyclic graph (DAG) (Fig. S1). We included sex (male, female), age at Visit 2 (years), parental highest education level (high school or below, greater than high school), nativity (US-born in 50 states/DC, born in a US territory or foreign country), current highest education level (high school or below, greater than high school), household income (< $30,000, ≥ $30,000), smoking status at Visit 2, and blood cell-type proportions at Visit 2. Sex, age, parental highest education level, and nativity served as baseline confounders. Current education level and household income were intermediate confounders, given that they were linked to adulthood adversity and EAA but affected by childhood adversity.

### Life-course models

We considered four common types of life-course models, sensitive period, critical period, accumulation, and pathway models, to explain the observed effect of adversity on EAA (Fig. S2).^25^ Both sensitive period and accumulation models assume childhood and adulthood are independent windows for adverse experiences to occur and contribute to EAA. The sensitive period model suggests both childhood and adulthood adversity contribute to EAA, but one of them contributes more. In the extreme case when the association between adversity and EAA is fully explained by either childhood or adulthood adversity, it would also be called the critical period model. When childhood and adulthood adversity contribute equally to EAA, it suggests the accumulation model. The pathway model considers adversity a chained exposure, in which childhood adversity could affect EAA by increasing the likelihood of exposure to adulthood adversity. In other words, the effect of childhood adversity is fully or partially mediated by adulthood adversity.

### Statistical analysis

**Fig. 1.**
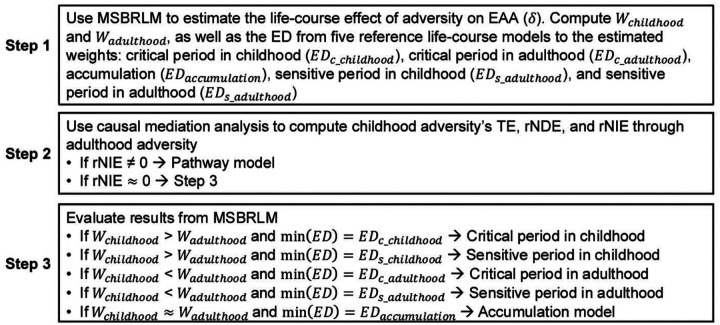
The pipeline for identifying the most compatible life-course model for psychosocial adversity Abbreviation: EAA, epigenetic age acceleration; ED, Euclidean Distance; min(*ED*), the smallest ED; MSBRLM, marginal structural Bayesian relevant life-course model; rNDE, randomized interventional analogue of natural direct effect; rNIE, randomized interventional analogue of natural indirect effect; TE, total effect; *W*_*adulthood*_, estimated weight in adulthood; *W*_*childhood*_, estimated weight in childhood Note: In step 1, the critical period in childhood is defined as (*W*_*childhood*_ = 1, *W*_*adulthood*_ = 0), critical period in adulthood (*W*_*childhood*_ = 0, *W*_*adulthood*_ = 1), accumulation (*W*_*childhood*_ = 0.5, *W*_*adulthood*_ = 0.5), sensitive period in childhood (*W*_*childhood*_ = 0.67, *W*_*adulthood*_ = 0.33), and sensitive period in adulthood (*W*_*childhood*_ = 0.33, *W*_*adulthood*_ = 0.67);

We followed the pipeline (Fig. 1) to estimate the effect of adversity and the most compatible life-course model for the observed data using the approaches described below. Briefly, we (1) estimated the life-course effect of psychosocial adversity on EAA and contributions of childhood and adulthood; (2) estimated the indirect effect of childhood adversity on EAA through adulthood adversity to evaluate the pathway model; and (3) evaluated the, critical period, sensitive period and accumulation model based on contributions of childhood and adulthood estimated by step (1) when minimal evidence of indirect effect was presented.

Current modeling approaches, such as the structural life-course modeling approach^47^ and the Bayesian relevant life-course model (BRLM),^48^ face a common limitation -- they cannot address intermediate confounding between later exposure and outcome.^49^ While the marginal structural model (MSM) can address this challenge,^33, 50^ it does not have a specific metric for measuring the compatibility between observed data and existing life-course hypotheses,^22^ which limits the ability to identify the best life-course model.

To address these limitations, we applied the BRLM^48^ under the MSM framework to propose a new implementation called MSBRLM (Supplemental Methods). Briefly, adversity was defined as the weighted sum of childhood and adulthood adversity. MSBRLM estimated the life-course association between adversity and EAA (δ), as well as the weights contributed by childhood (*W*_*childhood*_) and adulthood (*W*_*adulthood*_) adversity, as follows:

$$E(\text{EAA}|\text{Childhood},\text{Adulthood})=\beta_{0}+\delta (W_{\text{childhood}}*\text{Childhood}+W_{\text{adulthood}}*\text{Adulthood}),\text{where}\;{\text{W}}_{\text{childhood}}+W_{\text{adulthood}}=1.$$

Therefore, the model inherently assumes that the association in each period has the same direction (i.e., unidirectionality assumption). We first calculated inverse probability treatment weights (IPTW) for cumulative childhood and adulthood adversity to address measured confounding. Participants’ final weight was the product of their IPTW and HCHS/SOL sampling weight.^34^ To obtain normal-coverage 95% credible intervals (CrIs), we generated 200 Weighted Finite Population Bayesian Bootstrapping (WFPBB) samples to compute the posterior mean and 95% CrIs for all estimated parameters (Supplemental Methods).^51, 52^ Briefly, WFPBB reformed the target population by imputing the unobserved persons in the target population using observed participants based on their weights. A random sample of the same size as the analytic sample (N = 970) from the reformed target population, in which everyone had a weight of one, was used to fit the MSBRLM. We also calculated the posterior probability of δ > 0 (PP) as the strength of evidence that adversity would be associated with increased EAA. Under the assumption of exchangeability given measured confounders, the target population in this study would have experienced randomized psychosocial adversity in childhood and adulthood. Given consistency, no-interference, positivity, and unidirectionality assumptions, δ, *W*_*childhood*_, and *W*_*adulthood*_ can be interpreted causally (Supplemental Methods).

Next, we evaluated the pathway model by performing a causal mediation analysis for cumulative childhood adversity and EAA through cumulative adulthood adversity. Here, childhood-adulthood interaction was considered. To address the exposure-mediator confounding and mediator-outcome confounding affected by the exposure, we employed the MSM approach weighted by participants’ final weight to compute effect estimates with 95% confidence intervals (CIs) calculated via bootstrapping. Natural direct and indirect effects cannot be identified when the mediator-outcome confounding is affected by the exposure. Thus, we estimated the total effect (TE), and randomized interventional analogue of natural direct (rNDE) and indirect effects (rNIE) of childhood adversity to assess the compatibility with the pathway model. rNIE ≠ 0 would support the pathway model. The analysis was conducted using the *CMAverse* R package.

When rNIE ≈ 0, we further evaluated the compatibility with the sensitive period, critical period, and accumulation models. *W*_*childhood*_ = *W*_*adulthood*_ = 0.5 suggests the accumulation model, *W*_*childhood*_ > *W*_*adulthood*_ > 0 / *W*_*adulthood*_ > *W*_*childhood*_ > 0 sensitive period model, and *W*_*childhood*_ > *W*_*adulthood*_ ≈ 0 / *W*_*adulthood*_ > *W*_*childhood*_ ≈ 0 critical period model. The compatibility with the life-course models was also assessed by computing the posterior distribution of Euclidean Distance (ED) from five reference vectors of life-course models to the estimated weights: (1) critical period in childhood (*W*_*childhood*_ = 1, *W*_*adulthood*_ = 0), (2) critical period in adulthood (*W*_*childhood*_ = 0, *W*_*adulthood*_ = 1), (3) accumulation model (*W*_*childhood*_ =0.5, *W*_*adulthood*_ = 0.5), (4) sensitive period in childhood (*W*_*childhood*_ = 0.67, *W*_*adulthood*_ = 0.33), and sensitive period in adulthood (*W*_*childhood*_ = 0.33, *W*_*adulthood*_ = 0.67). The model with the smallest ED to the estimated weights was considered the most compatible.

In the secondary analysis, we assessed the effect of each adversity type (i.e., substance abuse by someone close, material deprivation, and abuse) on EAA and the most compatible life-course model using the same pipeline as the primary analysis. Several sensitivity analyses were performed. We used the well-established MSM approach weighted by participants’ final weight to estimate the effect of childhood adversity, adulthood adversity, and their additive effect (i.e., life-course effect), and checked whether the results were consistent with those from MSBRLM. Additionally, blood cell-type proportions and smoking status are known predictors of EAA and may be affected by psychosocial adversity. Therefore, we included (1) cell-type proportions and (2) smoking status in forming IPTW, separately, and reran MSBRLM, causal mediation analysis, and MSM, to check whether the associations were independent of the cell-type proportions and smoking status. Missing data were handled through multiple imputation (Supplemental Methods). All analyses were performed using R version 4.4.0.

## Results

A total of 970 Hispanic/Latino adults were included in the analyses (Table 1). The age range in years was 23–80 (mean [SD] = 44.7 [13.0]). Most of the adults were female (61.4%), born outside of the 50 states and territories in the US (81.4%), and had a high school diploma or higher as their highest education level (63.1%). More than half had the highest paternal and maternal education level in high school or above (55.3%). Fewer adults had a household income of $30,000 or higher (27.9%). Material deprivation was experienced by more than half of the adults in both childhood (54.2%) and adulthood (52.0%) (Table S3). Correlations among adversity types in childhood were relatively stronger compared to those in adulthood (Fig. S3).

### Life-course association between adversity and EAA

**Fig. 2.**
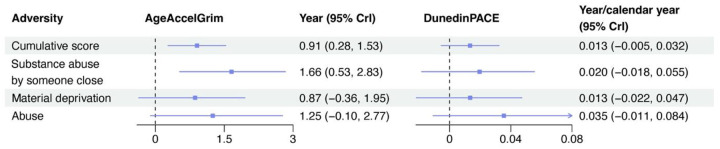
The life-course association between psychosocial adversity and epigenetic age acceleration (EAA) assessed by the Marginal Structural Bayesian Relevant Life-course Model (MSBRLM)

Every one-unit increase in the cumulative adversity score was associated with a 0.91-year (95% CrI: 0.28, 1.53, PP = 0.997) increase in AgeAccelGrim (Fig. 2A). With weaker evidence, adults with a one-unit higher life-course adversity score had a 0.013-year/calendar year (95% CrI: −0.005, 0.032, PP = 0.921) increased DunedinPACE.

For adversity types, substance abuse by someone close had stronger evidence in the association with increased AgeAccelGrim (δ = 1.66 years, 95% CrI: 0.53, 2.83, PP = 0.998) compared to material deprivation (δ =0.87 years, 95% CrI: −0.36, 1.95, PP = 0.932) and abuse (δ =1.25 years, 95% CrI: −0.10, 2.77, PP = 0.966) from childhood to adulthood. Imprecisely, abuse was also associated with an increased DunedinPACE (δ = 0.035 years/calendar year, 95% CrI: −0.011, 0.084, PP = 0.942), but estimates of the association of the substance abuse by someone close (δ = 0.020 years/calendar year, 95% CrI: −0.018, 0.055, PP = 0.850) and material deprivation (δ = 0.013 years/calendar year, 95% CrI: −0.022, 0.047, PP = 0.778) with DunedinPACE were modest.

### Compatibility with the pathway model

**Fig. 3.**
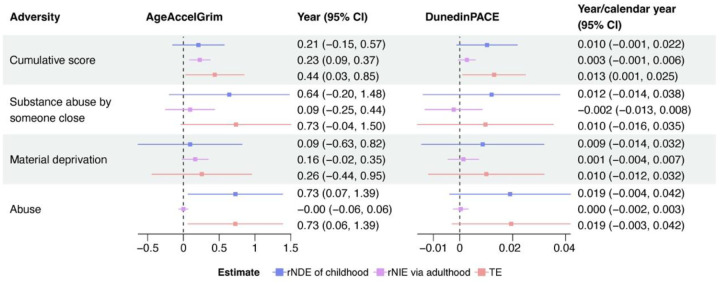
Causal mediation analysis for childhood adversity and EAA Abbreviation: rNDE, randomized interventional analogue of natural direct effect; rNIE, randomized interventional analogue of natural indirect effect; TE, total effect

The causal mediation analysis shows that the TE of a one-unit increase in the cumulative score of childhood adversity on AgeAccelGrim was 0.44 years (95% CI: 0.03, 0.85), with the rNIE of 0.23 years (95% CI: 0.09, 0.37) through cumulative adulthood adversity (Fig. 3). The TE of a one-unit increase in the cumulative childhood adversity score on DunedinPACE was 0.013 years/calendar year (95% CI: 0.001, 0.024), with a minimal rNIE of 0.003 years/calendar year (95% CI: −0.001, 0.006). Although imprecise, childhood material deprivation indirectly contributed to AgeAccelGrim through adulthood (rNIE = 0.16 years, 95% CI: −0.02, 0.35). Minimal and imprecise rNIE were found in other types of childhood adversity on AgeAccelGrim and all types of adversity on DunedinPACE. Therefore, the pathway model was the most compatible model explaining the association between cumulative adversity score, as well as material deprivation, and AgeAccelGrim (Table 2).

### Compatibility with the critical period, sensitive period and accumulation models

**Fig. 4.**
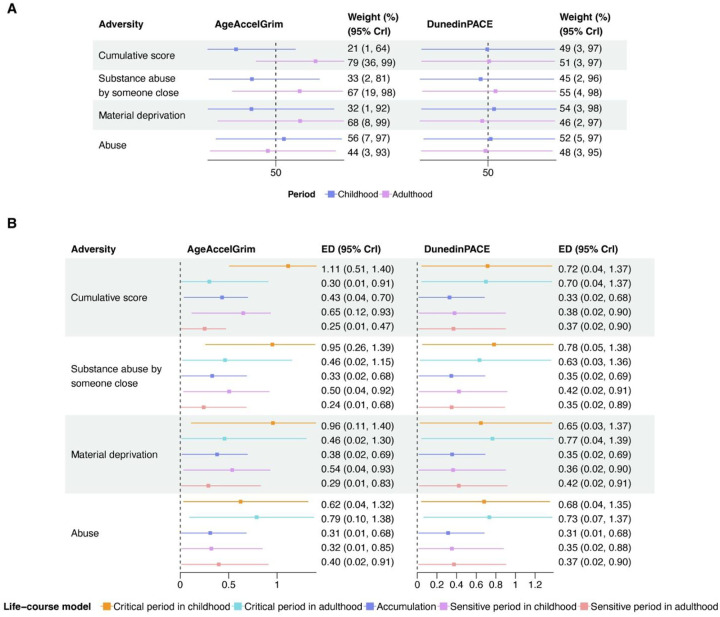
Weights and Euclidean Distance (ED) estimated by MSBRLM (A) The estimates of weights for childhood and adulthood; (B) The ED from reference life-course models to estimated weights. Note: The ED reflects the compatibility between estimated weights for childhood (*W*_*childhood*_) and adulthood (*W*_*adulthood*_) and referent life-course models, including critical period in childhood (*W*_*childhood*_ = 1, *W*_*adulthood*_ = 0), critical period in adulthood (*W*_*childhood*_ = 0, *W*_*adulthood*_ = 1), and accumulation (*W*_*childhood*_ = 0.5, *W*_*adulthood*_ = 0.5). A smaller ED indicates higher compatibility with the corresponding life-course model.

Supported by the pathway model previously, the cumulative adversity score (*W*_*adulthood*_ = 79%, 95% CrI: 36%, 99%) and material deprivation (*W*_*adulthood*_ = 68%, 95% CrI: 8%, 99%) in adulthood contributed a larger weight to the life-course association with AgeAccelGrim compared to their childhood counterparts (Fig. 4A, Table 2). Substance abuse by someone close (*W*_*adulthood*_ = 67%, 95% CrI: 19%, 98%) had a larger weight contributing to the life-course association, whereas childhood abuse had a similar weight compared to adulthood abuse (*W*_*childhood*_ = 56%, 95% CrI: 7%, 97% vs. *W*_*adulthood*_ = 44%, 95% CrI: 3%, 93%). The smallest ED for the association between substance abuse by someone close and AgeAccelGrim was from the sensitive period in adulthood (*ED*_*s_adulthood*_ = 0.24, 95% CrI: 0.01, 0.68), whereas the smallest ED for abuse was from the accumulation model (*ED*_*accumulation*_ = 0.31, 95% CrI: 0.01, 0.68) (Fig. 4B, Table 2).

For DunedinPACE, the weights for childhood (*W*_*childhood*_ = 45% ~ 54%) and adulthood (*W*_*adulthood*_ = 46% ~ 55%) were similar for the cumulative adversity score and across three types of adversity (Fig. 4A, Table 2). The smallest ED for associations of cumulative adversity score and adversity types were all from the accumulation model (Fig. 4B, Table 2).

### Sensitivity analysis

Using the MSM, estimates of the life-course association were very similar to those derived from MSBRLM (Fig.s S4 and S5). The estimated weight at each period was also generally consistent (*W*_*adulthood*_ > *W*_*childhood*_ in cumulative adversity score, substance abuse by someone close, and material deprivation for AgeAccelGrim; *W*_*adulthood*_ ≈ *W*_*childhood*_ in cumulative adversity score and abuse for DunedinPACE), except for substance abuse by someone close and material deprivation on DunedinPACE. Given the weak estimated association with large variance of these two adversity types, MSBRLM produced *W*_*adulthood*_ ≈ *W*_*childhood*_, but MSM estimated apparently larger weights in one period than another. Including cell-type proportions in the IPTW had little impact on the results from MSBRLM, causal mediation analysis, and MSM (Fig.s S6, S7, and S8). As expected, the life-course association of adversity estimated by MSBRLM and MSM with EAA, particularly with AgeAccelGrim, was mitigated after accounting for smoking status (Fig.s S9 and S10). The indirect effect of childhood adversity on AgeAccelGrim remained after accounting for smoking status (Fig. S11).

## Discussion

In this study, we found that the cumulative adversity score from childhood to adulthood was associated with increased AgeAccelGrim, and a less precise but consistent result was shown in DunedinPACE. The results when considering experiences of abuse followed the same direction but were imprecise for both epigenetic age measures. Substance abuse by someone close was associated with increased AgeAccelGrim but not DunedinPACE, aligning with findings in material deprivation; however, they showed greater uncertainty. When evaluating the life-course model, the pathway model was most compatible with the life-course association of cumulative adversity score and material deprivation with AgeAccelGrim, and the sensitive period in adulthood with substance abuse by someone close. In contrast, the accumulation model aligned most with cumulative adversity, substance abuse by someone close, and material deprivation in relation to DunedinPACE. The accumulation model was most compatible with the life-course association between abuse and both epigenetic age measures.

Cumulative adulthood adversity and most types of adversity had a larger direct contribution to AgeAccelGrim than their childhood counterpart, which is supported by studies showing that more recent stress exposure has a stronger impact on health outcomes.^53, 54^ Therefore, preventing adulthood adversity may effectively diminish the adverse impact of childhood adversity on biological aging. However, given the non-negligible indirect effect of cumulative childhood adversity and material deprivation through their adulthood counterpart, we suggest that the pathway model, rather than the sensitive period in adulthood, better explains their life-course pattern on AgeAccelGrim.

Preventing childhood adversity may block its direct impact on biological aging and lower the risk of adulthood adversity and its subsequent detriment. Previous studies generally suggest that persons who experience childhood adversity are more likely to re-experience adverse experiences in adulthood, supporting the idea of “chained exposure” in the pathway model.^55, 56^ Therefore, we may overlook the essential role of childhood by concluding adulthood to be a sensitive period without considering the mediating relationship. Abuse is an exception, showing a meaningful estimated effect on both AgeAccelGrim and DunedinPACE, with a similar contribution in both life stages. Abuse represents a threatening and traumatizing experience that may influence a person’s aging trajectory through distinct pathways compared to other types of adversity.^57^ However, we did not find evidence that adult abuse mediated the effect of child abuse, which is inconsistent with the expectation that persons who experience child abuse are highly likely to be re-traumatized later in life.^55, 56^ This could be due to the low prevalence of adult abuse in this HCHS/SOL subsample, limiting the correlation between abuse in childhood and adulthood.

The magnitude of the association between adversity and biological aging, and the corresponding most compatible life-course model, depended on the EAA measures, which capture different aspects of aging: GrimAge was trained to predict mortality,^44^ while DunedinPACE targets the rate of physiological decline in multi-organ systems.^45^ The estimate of life-course adversity in DunedinPACE is less pronounced than in AgeAccelGrim, and the estimates of substance abuse by someone close and material deprivation in DunedinPACE are minimal and imprecise. This HCHS/SOL subsample generally had a faster rate of aging than the reference population used to derive DunedinPACE (i.e., white men from New Zealand aged 45), with little variability (mean [SD] DunedinPACE = 1.08 [0.12]), which may explain the less pronounced results in DunedinPACE. The most compatible life-course model for the above adversity measures differed based on the clock: the pathway model or the sensitive period in adulthood for AgeAccelGrim, but the accumulation model for DunedinPACE. Similar results are found in Suglia et al., showing a larger contribution of adulthood stress to AgeAccelGrim compared to childhood stress, but a similar contribution when examining DunedinPACE.^22^ This suggests that adversity during different periods may affect biological aging through different mechanisms. Adulthood adversity may affect aging through pathways more directly linked to midlife mortality risk, such as lifestyle factors. Our sensitivity analysis shows that the life-course association of adversity with EAA decreased after accounting for smoking status, but the childhood contribution increased. In Suglia et al., they additionally adjusted for smoking in the analysis and found that the association of adulthood stress with AgeAccelGrim attenuated substantially and to a greater extent than the association with childhood stress, but with minimal changes in the association with DunedinPACE. Petrovic et al. also found that the association of adulthood financial difficulty on AgeAccelGrim was mostly mediated by lifestyle factors, whereas the proportion is small for childhood financial conditions.^24^ In contrast, chronic stress from childhood adversity may already be biologically embedded and accelerate physiological decline to a similar extent as adulthood adversity.^58, 59^ The impact may not be immediately exhibited as an increased mortality rate in our population, in which participants were generally in midlife. Life-threatening chronic conditions may still be progressing at this time and increase the mortality risk when the population grows older.

The estimates from the MSBRLM and MSM are very similar regarding life-course associations. The contributions from childhood and adulthood estimated from these two models are also consistent when the corresponding life-course effects are pronounced or precise. These support the potential of MSBRLM being a reliable tool in life-course epidemiology by incorporating advantages in both the existing life-course modeling approach and MSM. The inconsistent results in period-specific contributions given weak and imprecise life-course effects may stem from the different algorithms underlying these two models. However, it does suggest performing further simulation studies to evaluate the performance of MSBRLM as opposed to MSM when the data have large variance, given that MSBRLM has multiple metrics to help identify the most compatible model.^48, 60^

The study has several strengths. It is the first to evaluate the pattern of psychosocial adversity in different domains affecting biological aging under multiple life-course models. We proposed a framework incorporating a life-course modeling approach and causal mediation analysis that can comprehensively evaluate different life-course models that best explain the effect of exposure on outcomes. We also developed a new version of BRLM, MSBRLM, to allow adjustment for intermediate confounding, a common limitation of the current life-course modeling approach, and multi-metrics to evaluate models simultaneously. However, some limitations are listed below. First, childhood adversity was measured in adulthood, which presents a risk of misclassification. However, the misclassification is most likely to be independent and non-differential, given different tools to measure adversity and EAA and the prospective design. Second, we had limited types of adversity that happened in both childhood and adulthood in our population, which prevents us from generalizing the conclusion to other domains of adversity. Each type of adversity was also measured by a single item, limiting the reliability. Third, we used the cumulative score for childhood and adulthood adversity, which assumes that each adversity type contributes equally to the outcome. Alternative ways to quantify the cumulative exposure to adversity should be explored. Fourth, biological aging measured by epigenetic age markers in blood may reflect only cardiovascular aging and may not generalize to other organ systems. However, DunedinPACE uses blood DNAm to predict biomarkers across multiple organ systems, which may improve generalizability. Fifth, MSBRLM is computationally intensive and requires significant computational resources to execute. MSBRLM has also not been tested for its performance and should be examined through future simulation studies. However, the model generally yielded consistent results with conventional MSM, which supports its reliability. Finally, the exchangeability assumption may be violated due to residual confounding given the observational design, but we tried to reduce the risk of uncontrolled confounding by adjusting for multiple baseline and intermediate confounders.

## Conclusion

Life-course psychosocial adversity may accelerate biological aging. Although adulthood adversity shows a stronger association with biological age acceleration, childhood adversity may still be an important target for intervention, as it could indirectly influence biological aging by increasing the likelihood of adulthood adversity. Psychosocial adversity may affect biological aging through distinct pathways, and the effects may be heterogeneous by type of adversity, which warrants future exploration. Future studies are also warranted to extend the measures of psychosocial adversity to encompass broader life periods (e.g., childhood, adolescence, early adulthood, midlife, and late life) and to include a wider range of adversity types (e.g., neglect, neighborhood stress, and low socioeconomic position) to more precisely and comprehensively understand the life-course effect of adversity on aging.

## Supplementary Material

Supplementary Files

This is a list of supplementary les associated with this preprint. Click to download.

- SupplementarymaterialsSOLLAEAA.docx

## Figures and Tables

**Table 1.** Study characteristics of the HCHS/SOL subsample

Characteristics	N = 970 n (%)^a^
Age (years) at Visit 2, mean (SD)	50.50 (12.91)
Age (Years) range at Visit 2, min, max	23, 80
Sex	
Female	596 (61.4)
Nativity	
US-born	180 (18.6)
Parental highest education level	
High school or above	477 (55.3)
Highest education level	
High school of above	611 (63.1)
Yearly household income	
$30,000 or more	255 (27.9)
Smoking status at Visit 2	
Never	599 (61.8)
Former	207 (21.3)
Current	164 (16.9)
AgeAccelGrim at Visit 2, mean (SD)	0.00 (3.90)
DunedinPACE at Visit 2, mean (SD)	1.08 (0.12)

a The proportion may not sum up to be 1 due to missing values

**Table 2.** Summary of the most compatible life-course model for psychosocial adversity

	rNIE ≠ 0	*W*_*childhood*_ vs. *W*_*adulthood*_	Smallest ED	Most compatible model
**AgeAccelGrim**				
Cumulative score	Yes	*W*_*childhood*_ < *W*_*adulthood*_	*ED*_*s_childhood*_	Pathway model
Substance abuse by someone close	No	*W*_*childhood*_ < *W*_*adulthood*_	*ED*_*accumulation*_	Sensitive period in adulthood
Material deprivation	Yes	*W*_*childhood*_ < *W*_*adulthood*_	*ED*_*accumulation*_	Pathway model
Abuse	No	*W*_*childhood*_ ≈ *W*_*adulthood*_	*ED*_*accumulation*_	Accumulation model
**DunedinPACE**				
Cumulative score	No	*W*_*childhood*_ ≈ *W*_*adulthood*_	*ED*_*accumulation*_	Accumulation model
Substance abuse by someone close	No	*W*_*childhood*_ ≈ *W*_*adulthood*_	*ED*_*accumulation*_	Accumulation model
Material deprivation	No	*W*_*childhood*_ ≈ *W*_*adulthood*_	*ED*_*accumulation*_	Accumulation model
Abuse	No	*W*_*childhood*_ ≈ *W*_*adulthood*_	*ED*_*accumulation*_	Accumulation model

Abbreviation: ED, Euclidean distance; rNIE, randomized interventional analogue of natural indirect effect; *W*_*childhood*_, the estimated weight for childhood; *W*_*adulthood*_, the estimated weight in adulthood

## Data Availability

The data are available upon request from the HCHS/SOL website http://www.cscc.unc.edu/hchs/
